# Glucose-regulated protein 94 (Grp94/gp96) in viral pathogenesis: Insights into its role and therapeutic potentials

**DOI:** 10.1016/j.ejmech.2025.117713

**Published:** 2025-04-30

**Authors:** Hao Xu, Brian S.J. Blagg

**Affiliations:** Department of Chemistry and Biochemistry, Warren Center for Drug Discovery, The University of Notre Dame, 305 McCourtney Hall, Notre Dame, IN, 46556, USA

**Keywords:** Glucose-regulated protein 94 (Grp94/gp96), Viral infection, Immunomodulation, Small molecule inhibitors, Anti-Viral agents

## Abstract

Glucose-regulated protein 94 (Grp94/gp96) is endoplasmic reticulum (ER) resident form of the 90 kDa heat shock protein 90 (Hsp90) that is responsible for folding, maturation and stabilization of more than 400 client proteins. Grp94 has been implicated for various diseases including metastatic cancer, primary open-angle glaucoma, and infectious diseases. In fact, Grp94 plays critical roles in different stages of viral infection cycle. It chaperones receptor proteins and viral glycoproteins that are necessary for viral entry and replication. Beyond its role in protein homeostasis, Grp94 modulates host cellular processes such as apoptosis and immune responses, which are often exploited by viruses to sustain infection. This work provides an overview of the roles of Grp94 in viral pathogenesis across various viruses and its involvement in immune modulation with the development of Grp94-selective inhibitors and their potential as anti-viral therapeutics.

## Graphical Abstract

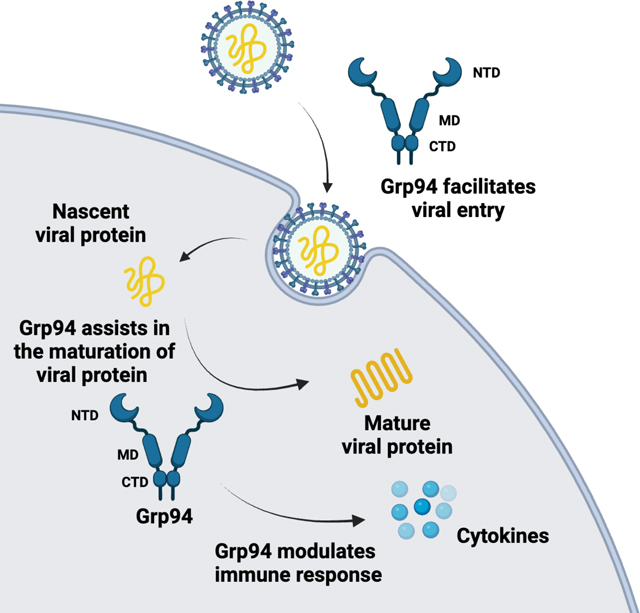

## 1. Introduction

Heat shock proteins (Hsps) are a family of molecular chaperones that are ubiquitously expressed in almost all living organisms and are upregulated in response to stressful conditions such as heat, malignant transformation and infection [1–3]. Mammalian Hsps are classified into groups based on molecular weight such as Hsp40, Hsp70, and Hsp90, among which Hsp90 is among the most abundant protein in eukaryotes and has been widely studied [4,5]. Hsp90 and its co-chaperones maintain cellular homeostasis by promoting the folding and maturation of newly synthesized proteins. It is required for the conformational maturation of regulatory proteins including kinases, transcription factors and steroid hormone receptors, which are essential for signal transduction, cell cycle control and apoptosis, respectively [6–8]. Hsp90 function is tightly regulated by ATPase activity, and inhibition of Hsp90’s ATPase activity has been studied in various diseases, particularly in cancer and infectious disease. Notably, Hsp90 has shown to play an important role in viral pathogenesis [9,10].

Hsp90 constitutes 1–2 % of the total cellular protein under non-stressed conditions and exists as four isoforms. The inducible and constitutively expressed isoforms, Hsp90α and Hsp90β, respectively, are found in cytosol; glucose regulated-protein 94 (Grp94) is localized in endoplasmic reticulum (ER); and tumor necrosis factor receptor-associated protein 1 (TRAP1) resides in mitochondria. Hsp90 functions as a homodimer under normal cellular conditions with each monomer consisting of an N-terminal domain (NTD), a middle domain (MD), and a C-terminal domain (CTD). The N-terminal domain contains a unique ATP-binding site which allows for the development of selective inhibitors [11]. In fact, Hsp90 also forms homo-oligomers and epichaperome hetero-oligomers dictated by the environment [12]. Unfortunately, more than 20 Hsp90 N-terminal inhibitors have entered clinical trials, but most have failed due to dose-limiting toxicities [13]. One of factors that contributes to these detriments is pan-inhibition of all four isoforms leading to induction of the heat shock response (HSR) and elevated levels of Hsp90. Pan-inhibition of Hsp90 stimulates the release of heat shock factor 1 (HSF-1), which then trimerizes and becomes phosphorylated before translocation into the nucleus, wherein it activates transcription of heat shock proteins including Hsp90 [14,15]. In addition, cardio-, ocular-, and hepatoxicities have been observed for many of these pan-inhibitors [16]. Therefore, the development of an alternative approach such as isoform-selective Hsp90 inhibition is highly desired.

Grp94, also known as gp96, has the most distinct ATP-binding site amongst all four isoforms and is only essential during embryonic development, but not developed cells [17,18]. Therefore, Grp94 represents a non-toxic target that can be pursued for the development of selective inhibitors. As a chaperone primarily localized in the ER, Grp94 is involved in the maturation of secretory and membrane-bound proteins, the regulation of Ca^2+^ homeostasis, and host defense via ERAD [18–21]. In fact, several viruses exploit the host’s ER chaperone machinery for the folding and assembly of their glycoproteins, which are critical for viral entry, replication and formation of infectious particles [9,22,23]. Viral infection hijacks the ERAD machinery and results in the accumulation of misfolded proteins in the ER lumen. Furthermore, viruses manipulate the host’s proteostasis network for the biogenesis of viral proteins, and chaperones such as Grp94 facilitate this process for the rapid production of multifunctional proteins [24,25]. For example, GRP94 is involved in the maturation of viral envelope glycoproteins in viruses such as hepatitis C virus (HCV) and dengue [26,27]. Therefore, inhibition of Grp94 has been implicated as a therapeutic target to treat viral infections.

**Fig. 1.**
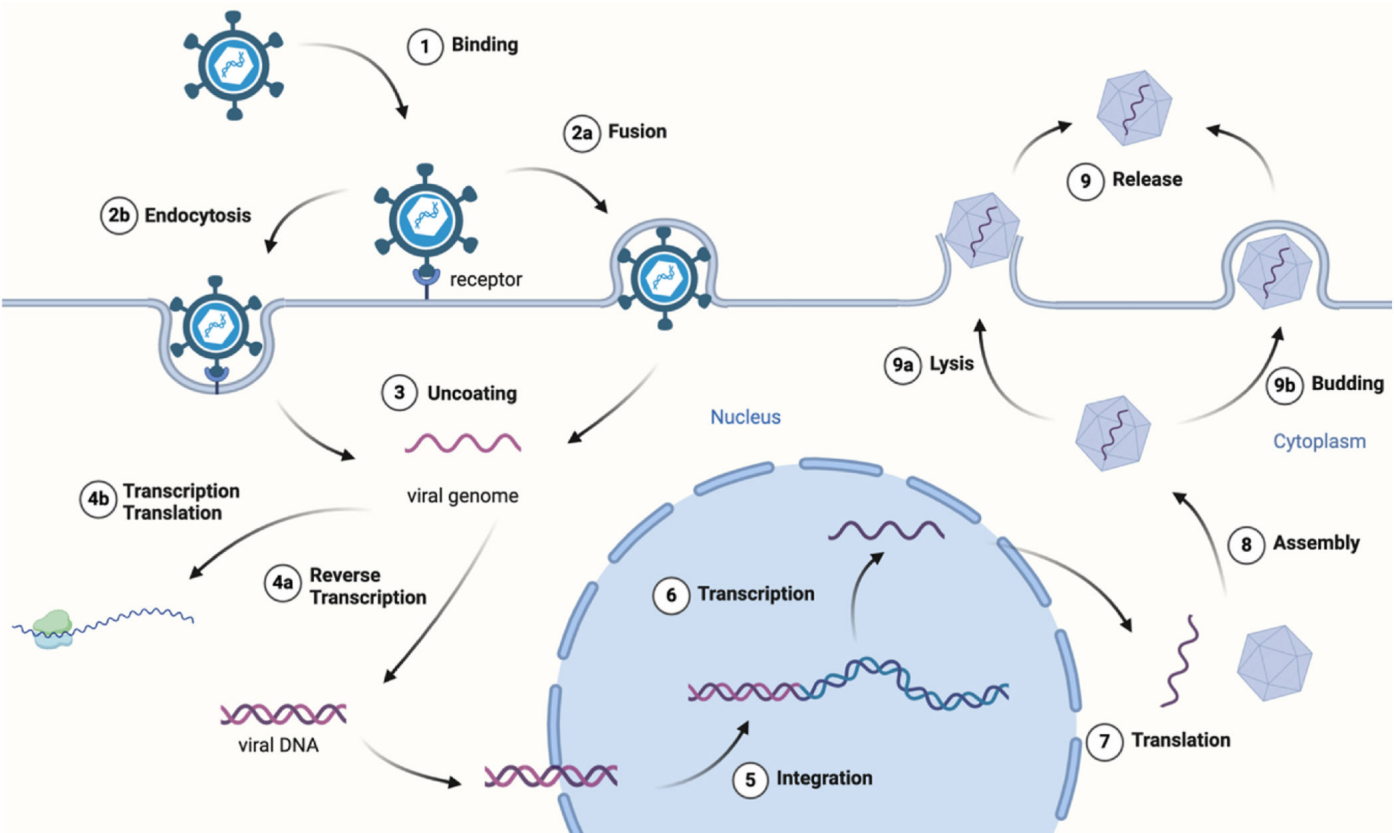
Viral infection cycle. The cycle begins with viral binding (1) and entry into the host cell via fusion (2a) or endocytosis (2b), followed by uncoating (3), genome replication, transcription, and translation using the host’s machinery (4–7). The newly synthesized viral components are then assembled into mature virions (8), which are released to infect new cells via lysis (9a) or budding (9b).

Viruses are intracellular parasites that depend upon the host machinery for protein synthesis, replication, and viral particle production. Viruses are classified into two groups: enveloped viruses wherein they contain a lipid bilayer surrounding their capsid; and non-enveloped viruses that lack a lipid envelope and consist only of the protein capsid [28]. To cause infection (Fig. 1), viruses attach to cell surface receptors and enter the host cells via internationalization including fusion and receptor-mediated endocytosis. Grp94 acts as a coreceptor by chaperoning receptors such as the low-density lipoprotein receptor (LDLR), which facilitates entry by enveloped viruses such as human herpesvirus 6 (HHV-6) and vesicular stomatitis virus (VSV) [29,30]. The viral capsid is then uncoated to release the viral genome, which undergoes transcription and translation. The newly manufactured viral genome and proteins are packaged into virions that are released via lysis or budding to propagate infection. Studies have shown that Grp94 associates with hepatitis B virus (HBV) polymerase, and Grp94 knockout inhibits dengue virus 2 (DENV2) replication [31,32]. Grp94 plays a critical role during the infection as well as the immunological response. This review aims to present the diverse function played by Grp94 throughout the infectious cycle and in the immune system. In addition, the development of Grp94-seletive inhibitors for the treatment of viral infections will also be discussed.

## 2. Hsp90 folding machinery and Grp94 structure

**Fig. 2.**
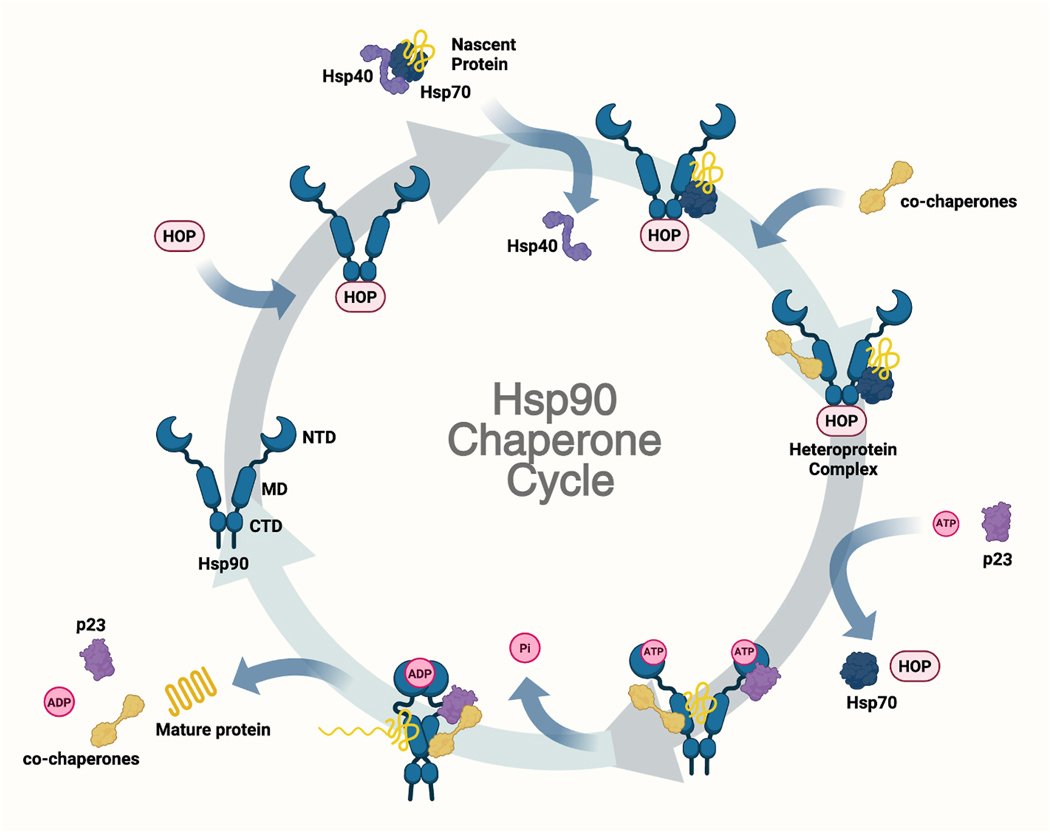
Hsp90 chaperone cycle. The cycle begins with the open state, where nascent protein and co-chaperones bind, followed by ATP binding and hydrolysis that induces a closed conformation, allowing proper folding, and leading to mature protein release and resetting of the cycle.

The Hsp90 chaperone cycle begins with a nascent polypeptide delivered to Hsp90 by the Hsp70/Hsp40 complex (Fig. 2) [33]. Upon delivery, co-chaperones interact with the Hsp90 dimer to form an activated heteroprotein complex that assists in correct folding of the polypeptide substrate. ATP hydrolysis at the NTD and p23 association promotes maturation and release of the client proteins [33–35]. The Hsp90 heteroprotein complex then dissociates and the Hsp90 dimer is available to repeat the cycle [36]. However, when an inhibitor binds the N-terminus of Hsp90, the client protein does not reach maturity and is instead ubiquitinated and degraded via the proteasome. In fact, the NTD of Hsp90 contains residues susceptible to post-translational modifications (PTMs), such as glycosylation, that can alter Hsp90 conformations [12]. For example, N-glycosylated occurs at Asn217 on Grp94 under normal conditions; whereas, malfunctional Grp94 is glycosylated at Asn62, which results in formation of the epichaperome and alters the cellular localization of Grp94 from the endoplasmic reticulum to the plasma membrane [37].

Similar to other isoforms of Hsp90, the NTD of Grp94 contains a Bergerat fold that is a druggable and unique ATP-binding site [38]. However, the ATP-binding sites of all four isoforms share >85 % identity, posing a significant challenge for the development of selective inhibitors [36]. Notably, Grp94 contains a 5 amino acid insertion (QEDGQ) into its primary sequence that is not present in other Hsp90 isoforms, which results in a secondary binding pocket (Site 2) found exclusively in Grp94 [39,40]. In fact, Grp94 contains 3 sites (Site 1, 2 and 3) that remain closed while in the apo form, but open during conformational changes to the NTD lid region in response to ligand binding. Site 1 is present in all four isoforms, whereas Site 3 is the outermost site wherein ligands bind to site 3 and exhibit energetically unfavorable conformations, thus making site 2 particularly important for both binding and selectivity [41,42]. In the apo form, the Grp94 site 2 is blocked by Phe199. Access to site 2 requires the conformational adjustment of Tyr200 and the accompanying helices to displace the amide backbone and rotate Phe199 25° [32,43,44]. Conformational changes to open a similar site 2 in other isoforms have not been observed, and therefore, site 2 represents an opportunity to develop Grp94-selective inhibitors.

## 3. Role in virus infectious cycle

### 3.1. Viral entry

A critical step in viral entry requires attachment of the virus to the receptor located on the host cell membrane, wherein Grp94 supports viral entry by maintaining cell surface receptors. Integrins have been identified as Grp94-dependent client proteins, and integrins control binding and the uptake of extracellular vesicles and viruses [45,46]. Therefore, Grp94 plays a crucial role in integrin-mediated attachment or entry of several enveloped viruses such as West Nile virus (WNV), Japanese encephalitis virus (JEV) and Zika virus [47–49]. In addition, multiple researchers have demonstrated that vesicular stomatitis virus (VSV) requires Toll-like receptors (TLRs) and LDL receptors (LDLRs), and cells that lack functional TLRs and LDLRs do not bind to the envelope glycoprotein of VSV [18,50]. In fact, TLRs and LDLRs are Grp94-dependent client proteins, suggesting that Grp94 is essential for the entry of VSV [51].

Grp94 interacts with or assists in the folding of enveloped viral glycoproteins that are critical for viral attachment and fusion with the host cell membrane [29]. Most glycoproteins bind to one or more cellular factors, and form complexes that act as ligands for receptor-mediated viral entry. Prusty et al. were the first to use proteomics to show that Grp94 is expressed on the cell surface and upon infection with Human Herpesvirus 6A and 6B (HHV-6A and HHV-6B), it interacts with the virions via its C-terminal domain [52]. Ma et al. later demonstrated that extracellular Grp94 binds HHV-6 glycoprotein Q1 (gQ1), and this complex then binds to the CD46 or CD134 receptors for HHV-6A and HHV-6B entry, respectively. Knockdown of Grp94 or halting its expression with antibodies inhibit HHV-6 infection [29]. The herpes simplex type virus 1 (HSV-1) depends on the glycoprotein B (gB) to mediate fusion of the virus with the host cell membrane, which facilitates entry into the cell. Activation of the Grp94 promoter by the mutant form of gB suggests that Grp94 forms a direct and stable interaction with the HSV-1 gB, but not with the fully processed viral protein [53].

**Fig. 3.**
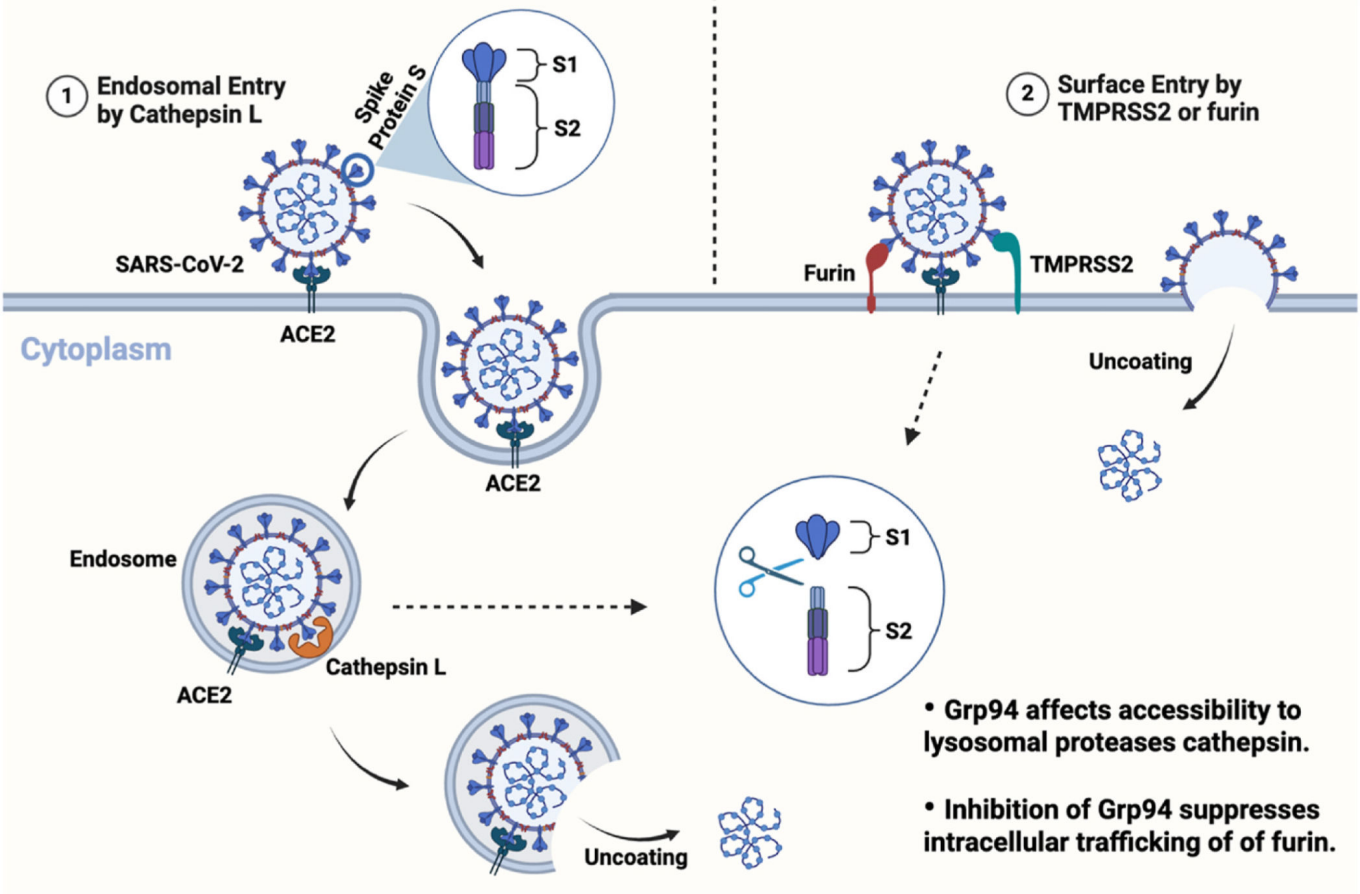
SARS-CoV-2 viral entry pathways. SARS-CoV-2 enters host cells by binding its spike protein (S) to ACE2 receptor on the cell surface, facilitating viral attachment. This interaction triggers proteolytic activation by host protease cathepsins L (1, left) or TMPRSS2/furin (2, right).

Grp94 also regulates cellular proteases that are necessary for virus binding. A notable example is the severe acute respiratory syndrome coronavirus 2 (SARS-CoV-2). The human angiotensin-converting enzyme 2 (ACE2) is the major cell surface receptor utilized for the entry of SARS-CoV-2 (Fig. 3) [54]. SARS-CoV-2 contains the enveloped spike protein S, which binds the ACE2 receptor and enters via fusion/endocytosis. Computational studies revealed a probable protective mechanism where Hsp70/Hsp90 forms stable complexes with S-ACE2 and/or angiotensin II (AngII), and reduces the availability of ACE2 for SARS-CoV-2 infection [55]. However, these findings need to be validated in cellular models. In contrast, the furin-like ACE2-binding spine on the S protein needs to be proteolytically activated by serine proteases at the S1/S2 cleavage site before the virus can penetrate the membrane and release viral genome [56–58]. These proteases include cell surface protease TMPRSS2, lysosomal proteases, cathepsins, and furin [59,60]. In fact, through association with complement C3, Grp94 directly affects accessibility to the lysosomal proteases, cathepsins [61]. Furthermore, Koo and coworkers demonstrated that Grp94 siRNA downregulates the cell surface levels of furin, and the administration of geldanamycin (GDA, an Hsp90 pan-inhibitor) led to decreased furin levels on the HEK293F cell surface, suggesting that inhibition of Grp94 suppresses intracellular trafficking of furin and its proteolytic activity at the cell membrane [62]. These data demonstrate that the Grp94 knockdown can decrease efficiency of SARS-CoV-2 viral entry by inhibiting the ACE2 receptor and cellular proteases such as furin and cathepsins.

### 3.2. Viral protein folding, replication and assembly

Viral glycoproteins are not only essential for mediating viral entry, but also serve as a target for immune response. Furthermore, overexpression of Grp94 enhances the replication of these viral glycoproteins. Hepatitis C virus (HCV) contains two envelope glycoproteins, E1 and E2, which are synthesized in the ER to form a heterodimer. E1 protein is important for viral entry, whereas E2 protein is the major target of neutralizing antibody response and facilitates viral infection [26,63,64]. In 2008, it was discovered that the anti-apoptotic effect of the HCV E2 protein was driven by Grp94-mediated nuclear factor kappa B (NF-κB) activation and the expression of survival gene products [63]. The E2 protein prevents apoptosis induced by the host immune system via the overexpression of Grp94, and instead induces tumor-necrosis–related apoptosis-inducing ligand (TRAIL) which leads to the expression of anti-apoptotic proteins and promotes viral replication. Subsequent studies reported that the levels of transforming growth factor β1 (TGF-β1) were elevated in HCV infected hepatocytes in liver tissue derived from HCV patients, and HCV E2 protein degrades AIMP1/p43 before triggering the overproduction of TGF-β1 via Grp94 mediated NF-κB activation [65,66]. Inhibition of Grp94 can reduce anti-apoptotic activity and the production of TGF-β1 that is induced by the E2 glycoprotein and thus, clear viral infection. Moreover, expression of the pseudorabies virus (PRV) glycoprotein B (gB) significantly increased the expression of Grp78/Bip and Grp94, and upregulated phosphorylation of eIF2α in the PERK pathway in BHK-21 cells [67]. Reduced levels of phosphorylated eIF2α and PRV titer in siPERK-treated cells after PRV infection suggests that PRV replication benefits from activation of the PERK pathway. While Grp94 indirectly activates PERK, Grp94 chaperones PRV gB, which triggers activation of the PERK pathway and enhances viral replication.

Beyond viral envelope glycoproteins, Grp94 also plays a critical role in promoting replication and folding of other viral components through a quality control mechanism that removes misfolded viral proteins. Dengue virus (DENV) and Zika virus (ZIKA) are two major faviviruses that utilize the ER machinery for their viral protein production and assembly. Genome-scale RNAi and CRISPR/Cas-9 based screenings identified the Hrd1 complex as one of many host factors required for DENV and ZIKA viral replication [68–70]. The Hrd1 complex utilizes the ER-associated degradation (ERAD) pathway to remove misfolded viral proteins that accumulate in the ER lumen [71]. Rothan et al. reported that Grp94 is a critical component of the Hrd1 ubiquitin ligase complex, and Grp94 knockdown inhibits DENV2 replication of both envelop and viral proteins [72]. Their data suggest that Grp94 knockdown interrupts the protein degradation machinery through ERAD, which results in the accumulation of misfolded viral proteins. In addition, the overexpression of Grp94 (BmGrp94) can facilitate the proliferation of *Bombyx mori* nuclear polyhedrosis virus (BmNPV) via inhibiting apoptosis [73]. Mechanistically, Gpr94 inhibition increases the expression of PERK/ATF4/ERO1, H_2_O_2_ production, and ER calcium efflux, which results in apoptosis. Beyond apoptosis, a recent study with Senecavirus A (SVA) revealed that Grp94 is a key contributor to SVA replication by promoting ER stress-induced autophagy [74]. The Grp94 inhibitor HCP1 was found to suppress SVA replication by inhibiting Grp94 protein levels and the autophagy induced by SVA infection.

Grp94 associates with Hepatitis B virus (HBV) polymerase in human liver HepG2 cells [31,75]. Studies have demonstrated that Grp94 expression is elevated during the progression of HBV-induced diseases from chronic hepatitis to cirrhosis [76]. The binding of HBV polymerase to HBV εRNA dissociates Grp94 from HBV polymerase, indicating that Grp94 is required for the maturation and stabilization of HBV polymerase and responsible for maintaining its activity [31]. Mechanistically, activation of NF-κB pathway that is induced by HBV x protein (HBx) results in high Grp94 transcription levels, and elevated expression of Grp94 leads to enhanced HBV replication [77]. This evidence suggests that Grp94 promotes the production of viral proteins by eliminating misfolded proteins via the ERAD pathway, while associating with viral polymerases. In addition, Valk et al. identified Evi12 as a common viral integration site for retroviral insertion. When retroviruses integrate into the genome of the host cells, they insert within Evi12 region located upstream of the gene encoding for Trp1/Grp94, suggesting that Trp1/Grp94 is a target gene for retroviral activation [78].

## 4. Role in immune modulation

One of the properties manifested by Grp94 in immune response has been its use as an adjuvant [79–81]. Grp94 is involved in the cross-presentation of antigens, a process wherein antigen-presenting cells (APCs) such as dendritic cells and macrophages take up exogenous antigens from viruses that do not infect APCs and present them on major histocompatibility complex (MHC) class I molecules to activate CD8^+^ cytotoxic T lymphocytes (CTLs) [82,83]. Grp94 chaperones the peptides derived from these viruses and transports them to the APCs wherein the Grp94-peptide complex is internalized after bound to scavenger or toll-like receptors (TLRs) to trigger activation of the cross-presentation pathway [79–81]. The CD91 (LRP1) receptor is an example of a scavenger receptor recognized by the Grp94-peptide complex that mediates the internalization process. Interestingly, the interaction between CD91 and Grp94 was examined by Jockheck-Clark et al. [84] They found that Grp94-peptide cross-presentation was insensitive to CD91 ligands in the DC2.4 dendritic cell line, but was internalized by fluid-phase endocytosis instead of the receptor-mediated pathway.

Macrophages are key regulators of immune modulation and can be polarized into either pro-inflammatory (M1) or anti-inflammatory (M2) phenotypes to maintain immune homeostasis [85]. Pro-inflammatory cytokines trigger inflammation and help the host immune system defend viral infection. In fact, Grp94 closely interacts with macrophages to influence their function and development. Grp94 can interact with macrophage complement C3 and switch M2 macrophages towards a pro-inflammatory profile, which is dependent on IRE1α activation and functional Grp94 [61]. In this study, basal M2 expresses high levels of cell surface membrane Grp94 and complementary receptor 3 (CR3). Complement C3 is cleaved by cathepsin L intracellularly, producing C3b which is inactivated into the iC3b form. iC3b then binds the CR3 receptor and leads to an M2 anti-inflammatory phenotype. In contrast, under thapsigargin (Tg)-induced ER stress, Tg-treated M2 does not express membrane Grp94 due to inhibition of IL-4/STAT6. Instead, C3 binds to the C-terminal domain of Grp94. Upon co-secretion of the Grp94/C3 complex, cathepsin L cleaves C3 extracellularly, producing C3a and C3b, which bind to C3aR and CD46, respectively, to induce pro-inflammatory cytokines. These researchers later confirmed the involvement of Grp94 in the M2 pro-inflammatory profile and modulation of cathepsin L activity in obesity-related inflammation conditions [86]. As discussed, cathepsins are essential proteases for SARS-CoV-2 viral entry. In addition to regulating macrophage function, Grp94 is also important in macrophage development, which requires integrins to carry out fundamental processes such as adhesion and polarization. Studies have identified integrin αDβ2 as an adhesion receptor that is upregulated on macrophages to facilitate multiligand binding; integrin α2β1 modulates IL-4 and lipopolysaccharide which induce the M2 and M1 inflammatory phenotypes, respectively [87,88]. Notably, integrin αD, β2, and α2 subunits are Grp94 client proteins that rely upon Grp94 for proper folding and cell surface expression [89]. Of note, M2 lacks integrin β3 and produces increased levels of TGF-β1, which is associated with Grp94 mediated NF-κB activation during HCV infection as previously discussed [65,90]. These data provide a diverse function for Grp94 in viral entry and immune modulation against viral infection.

Grp94 is required for innate immune activation by interacting with toll-like receptors (TLRs), which depend upon Grp94 for the folding, trafficking and function, in particular TLR2, TLR4 and TLR9 [18]. TLRs are responsible for recognizing and responding to pathogen-associated molecular patterns (PAMPs), which includes lipopolysaccharides (LPS) and viral DNA [91]. Upon recognizing PAMPs, TLRs trigger downstream signaling cascades that involve adaptor proteins MyD88 or TRIF, ultimately leading to the activation of NF-κB and IRF3. Transcription factors induce the production of pro-inflammatory cytokines, and type I interferons, such as IFN-β [91]. Studies demonstrated that interactions between Grp94 and TLR2 or TLR4 produce a high level of pro-inflammatory cytokines and T cell activation, which amplifies the innate immune response [92]. Bernaleau et al. recently identified CCDC134 as an essential regulator of Grp94, and thus regulating the biogenesis of TLRs and the immune response [93].

## 5. Grp94-selective inhibitors and their therapeutic use as anti-viral agents

**Fig. 5.**
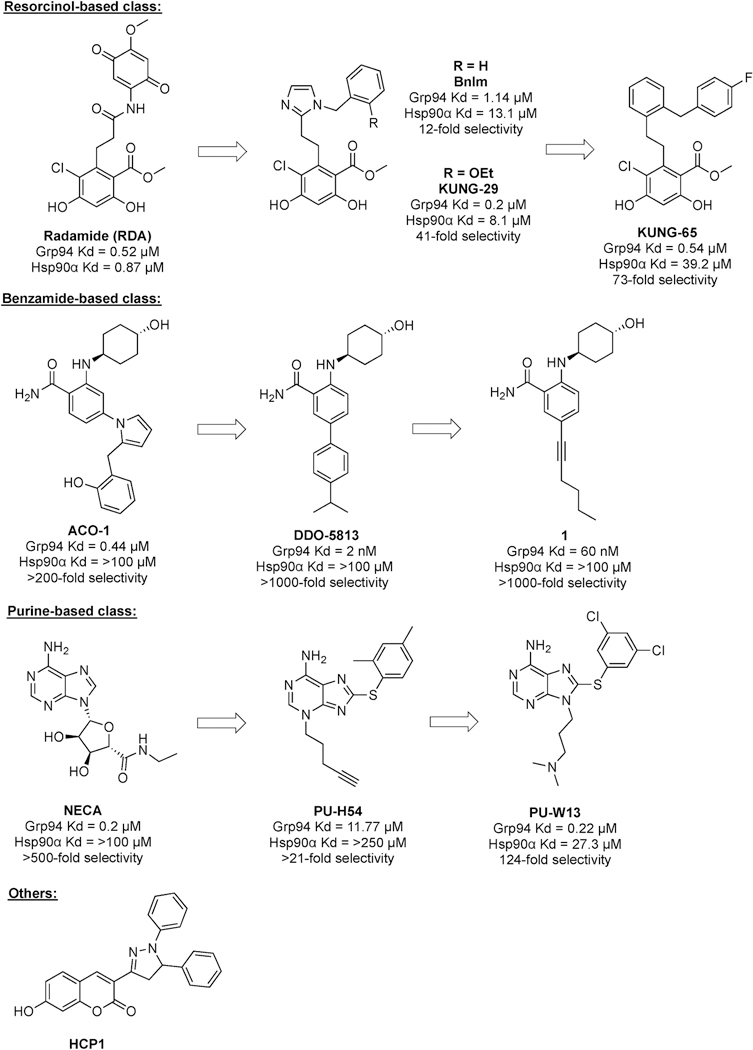
Three main classes of Grp94-selective inhibitors: resorcinol-based class, benzamide-based class, and purine-based class.

Grp94 has emerged as a promising target for the treatment of metastatic cancer and primary open-angle glaucoma, due to the lack of toxicity resulting from Grp94 inhibition and knockout in mammalian cells [18,94,95]. However, only a few studies have investigated the use of Grp94-selective inhibitors as anti-viral agents. Three main classes of Grp94-selective inhibitors have been developed thus far (Fig. 5). Radamide (**RDA**) was a chimera designed from the pan-Hsp90 inhibitors geldanamycin (**GDA**) and radicicol (**RDC**). However, replacement of the RDA amide with a *cis*-amide bioisostere (imidazole) yielded the first generation of resorcinol-based Grp94-selective inhibitors, from which **BnIm** was found to manifest 1.14 μM affinity for Grp94 and ~12-fold selectivity over Hsp90α. Subsequent SAR studies produced **KUNG29**, which contains an 2-ethyloxy benzyl chain and manifests 0.2 μM affinity and ~41-fold selectivity [17,96]. Introduction of a phenyl linker in lieu of the imidazole and the incorporation of a 4-fluorobenzyl side chain yielded the second generation of inhibitors, for which **KUNG65** was found to exhibit 0.54 μM affinity for Grp94 and ~73-fold selectivity [94]. A recent study has explored the ester group projected in the exclusive site 2 pocket in Grp94 with various amides [46]. In addition to resorcinolic inhibitors, benzamide-based Grp94-selective inhibitor **ACO1** (0.44 μM and >200 fold selectivity) was developed from the non-selective aminohexanol Hsp90 inhibitor, **SNX2112**. Xu and cow-orders discovered and optimized a benzamide inhibitor **DDO-5813**, which manifests 2 nM affinity for Grp94 and > 1000-fold selectivity. Replacement of the phenyl ring with a rigid alkyne gave compound **1**, which exhibited 60 nM affinity and improved PK properties [97,98]. **NECA** was the first identified purine-based Grp94-selective inhibitor, and subsequent work by the Chiosis lab resulted in the PU-based inhibitor, **PU-H54** (11.77 μM and >21 fold selectivity) [32,99]. Exploration on the linker between the purine and aryl moiety as well as adenine core led to **PU-WS13**, which manifests 0.22 μM and 124-fold selectivity [100]. In addition to these three main classes of Grp94 inhibitors, the Miao lab revealed **HCP1** as an Hsp90 inhibitor with Grp94 inhibitory activities [101,102].

As discussed, Grp94 knockdown inhibits DENV2 and ZIKA virus replication through inhibition of Hrd1 ubiquitin ligase-mediated ERAD. In the same study, researchers assessed the effect of **PU-WS13** on virus replication along with the Hrd1 ubiquitin ligase complex inhibitor, **CDDO-me** [72]. **PU-WS13** inhibits DENV2 and ZIKA infections with an EC_50_ ~27 nM and ~25 nM in Huh-7 cells, respectively, without anti-proliferative activity. **PU-W13** also inhibits late stage DENV2 replication as determined via time-of-addition experiments. Furthermore, the coumarin pyrazoline derivative **HCP1** upregulated the number of lipid droplets (LDs) and inhibited autophagy in vascular endothelial cells [103]. Based on these observations, Wang et al. investigated the effect of **HCP1** on Senecavirus A (SVA) replication, which modulates host cell apoptosis and autophagy to enhance the infection cycle [74]. SVA promotes replication by activating ER stress-induced autophagy through PERK and activating transcription factor 6 (ATF6). **HCP1** increased the levels of autophagy markers LC3II and p62 in SVA infected BHK-21 cells, indicating that **HCP1** disrupts autophagic flux. Meanwhile, the overexpression of Grp94 increased p62 protein, but had no influence on autophagic flux after the treatment of **HCP1**.

One of the most common uses of Grp94 is an adjuvant for viral vaccines, which consist of complexes between Grp94 and antigenic peptides [104–108]. Pishraft-Sabet and co-workers constructed a polytope (PT) DNA vaccine that contained HCV immunodominant T lymphocytes and B-cell epitopes, wherein the C-terminus of polytope was fused with the N-terminal domain of Grp94 (NTgp96) [104]. A significant T-cell and antibody response was observed in HCV infected mice upon treatment with the PT DNA vaccine fused with NTgp96 as compared to the DNA vaccine alone. Interestingly, this immunomodulatory effect was reduced when NTgp96 was fused to the N-terminus of HCV DNA polytope. Another group investigated the antiviral effect of Grp94 adjuvant vaccines in HBV transgenic mice [105]. Immunization with HBV surface (HBsAg) and/or core (HBcAg) antigens with Grp94 led to increased infiltration of CD8^+^ T cells and significantly decreased liver HBc levels as well as reduced viral load. Similarly, Niu et al. designed a multi-epitope-based vaccine that contains two peptides (AR16 and hPAB) and canine-Gpr94 for the treatment of Rabies virus [106]. The safety and efficacy of this vaccine was validated in mice and dogs wherein neutralizing antibodies were observed in both models. A recent study with SARS-CoV-2 utilized a novel vaccine that engineered Grp94 and SARS-CoV-2 Spike protein (gp96-Ig-S) with OX40L-Fc fusion proteins [107,108]. This vaccine induced activation of the SARS-CoV-2 S protein specific antibody. They also discovered that gp96-Ig-S significantly reduced metabolic and inflammatory B cells in old mice, indicating that it improves immune function in old mice as compared to young mice.

## 6. Conclusion and outlook

Grp94 plays an important role in different stages of viral infection, such as viral entry, viral replication, folding, and immune modulation. Grp94 supports the entry of numerous envelope and non-envelope viruses either independently or by assisting in the maturation of viral receptors/co-receptors. As a key ER chaperone, Grp94 assists in the folding and stability of viral glycoproteins to ensure their functional maturation and trafficking, which can be exploited by viruses to sustain infection and propagation. In addition, Grp94 facilitates viral replication by stabilizing essential host factors and viral complexes. Unlike intracellular Grp94 that supports viral infection, extracellular Grp94 modulates immune responses by facilitating cross-presentation of antigens and interacting with TLRs to strengthen both innate and adaptive immunity.

The mechanism by which Grp94 facilitates viral infection and immune regulation must be fully understood before Grp94 inhibitors can be utilized to treat viral infection. While the mechanism played by Grp94 during viral infection is complex, it is clear that Grp94 inhibition manifests significant anti-viral activity. Moreover, the immunomodulatory properties manifested by Grp94 highlights its use as a vaccine adjuvant or immunotherapeutic target during viral infection. However, much remains to be understood about Grp94-selective inhibitors on viral infection and the role played by Grp94 in antiviral immune response. Future research will most likely focus on the elucidation of virus specific processes that are dependent upon Grp94 and the benefits of inhibiting such processes.

## Figures and Tables

**Fig. 4.**
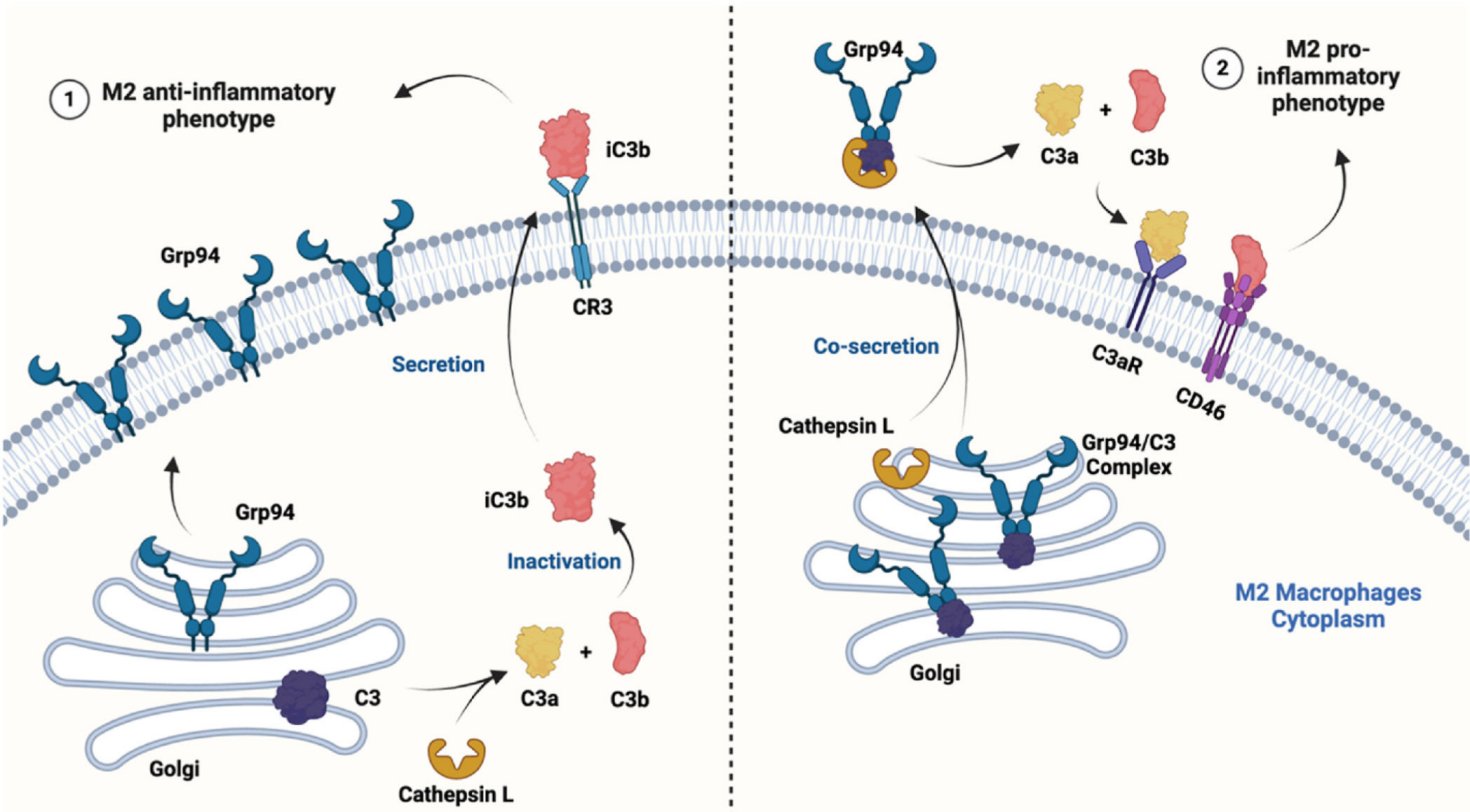
In basal M2 (left), C3 is cleaved by cathepsin L, and generates C3b which is inactivated to iC3b. iC3b binds receptor CR3, which contributes to M2 anti-inflammatory phenotype. In Tg-treated M2 (right), GRP94 forms complex with C3. Secreted Grp94/C3 complex is cleaved by extracellular cathepsin-L, producing C3b and C3a. Both contribute to the induction of M2 pro-inflammatory profile.

## Data Availability

Data will be made available on request.
